# Masson’s tumor of the kidney: a case report

**DOI:** 10.1186/s13256-018-1898-2

**Published:** 2018-12-22

**Authors:** Mohamed Ali Essid, Abderrazak Bouzouita, Ahlem Blel, Maroua Gharbi, Marouen Chakroun, Aycha Ben Miled, Haroun Ayed, Mohamed Cherif, Mohamed Riadh Ben Slama, Amine Derouiche, Mohamed Chebil

**Affiliations:** 1 Department of Urology, Charles Nicolle University Hospital of Tunis, Boulevard du 9 Avril 1938, 1006 Tunis, Tunisia; 2 Department of Pathology, Charles Nicolle University Hospital of Tunis, Tunis, Tunisia; 3 Department of Radiology, Charles Nicolle University Hospital of Tunis, Tunis, Tunisia

**Keywords:** Bosniak classification, Intravascular papillary endothelial hyperplasia, Kidney, Masson’s tumor, Nephrectomy, Renal cyst

## Abstract

**Background:**

Intravascular papillary endothelial hyperplasia (known also as Masson’s tumor) is a benign vascular lesion that commonly occurs in the skin and is rarely found in solid organs, especially in the kidney. In what follows, we will look into the first case of an unexpectedly diagnosed Masson’s tumor of the kidney presenting as a suspicious renal cyst.

**Case presentation:**

A 61-year-old Arab man presented with a left renal cyst, incidentally revealed by ultrasonography. The laboratory values were unremarkable. Computed tomography and magnetic resonance imaging demonstrated a 38 mm left renal midportion Bosniak IV cyst. Our patient underwent a radical nephrectomy. Histopathology revealed the diagnosis of intravascular papillary endothelial hyperplasia. There was no recurrence detected after 9 years of follow-up.

**Conclusions:**

Renal intravascular papillary endothelial hyperplasia is a rare benign tumor which can mimic a suspicious renal mass on radiological findings. Thus, this entity should be considered more often in the thick of the diagnostic possibilities in order to avoid unnecessary nephrectomies.

**Electronic supplementary material:**

The online version of this article (https://doi.org/10.1186/s13256-018-1898-2) contains supplementary material, which is available to authorized users.

## Background

Intravascular papillary endothelial hyperplasia (IPEH) is a benign vascular lesion commonly known as Masson’s tumor, which was first described in 1923 by Masson. IPEH is a reactive process of endothelial proliferation that takes place around thrombi in the setting of venous stasis.

This pathology occurs more often in the extremities of the body on cutaneous tissues. Only 12 cases of this tumor localized in the kidney have been described in the literature (Table 1). In this article, we report the first case in which this tumor presents as a suspicious renal cyst. We aimed to provide further insight about this rare entity to better characterize it, in order to avoid some unnecessary nephrectomies.

**Table 1:** Clinical findings of renal Masson’s tumor in the literature

	Year	Source	Age (year)	Sex	Presentation	Side	Size (mm)	Location	Radiologic findings	Preoperative diagnosis	Treatment	Follow-up (month)
1	1990	Garber *et al.* [6]	57	M	Hematuria	R	30	Midportion	CT: renal mass MRI: high intensity enhancing	Hypernephroma Vascular malformation	Nephrectomy	13/NED
2	1996	Steffee and Iskandar [12]	63	F	Acute renal failure	G	NA	Hilar	NA	Abnormal vascular flow	Transplant Nephrectomy	NA
3	1997	Johraku *et al*. [3]	55	F	Incidentally	R	30	Hilar	US: isoechoic mass CT/MRI: peripheral enhancement	Renal hemangioma Renal neoplasm	NSS	NA
4	2000	Kim *et al*. [4]	7	F	Hematuria	L	18	Midportion	US: hypoechoic mass CT: renal mass	Wilms tumor Neuroblastoma	Nephrectomy	NA
5	2002	Van den Bogaert *et al*. [13]	64	M	Incidentally	L	26	Hilar	US: hyperechoic lesion CT: heterogeneous enhancement MRI: T1 hypointense T2 hyperintense	Renal neoplasm	Nephrectomy	NA
6	2005	Akthar *et al*. [14]	40	M	Hematuria	L	24	Midportion	US: hyperechoic lesion CT: soft tissue mass	Renal neoplasm	Nephrectomy	NA
7	2005	Akhtar *et al*. [14]	48	M	Incidentally	L	30	Hilar	US/CT: renal mass	Renal neoplasm	Nephrectomy	NA
8	2009	Rizza *et al*. [7]	49	M	Massive retroperitoneal hemorrhage	L	55	Hilar	NA	NA	Nephrectomy	NA
9	2011	Pelosi *et al*. [8]	30	M	Incidentally	L	12	Hilar	CT: renal mass	Renal neoplasm	Nephrectomy	NA
10	2012	Mehta *et al*. [1]	48	M	NA	NA	22	NA	NA	Renal neoplasm	NSS	132/NED
11	2016	Alkan *et al.* [10]	50	M	NA	L	40	Hilar	CT: renal mass with calcifications and internal homogeneous enhancement in the delayed phase MRI: T1 hypointense T2 hyperintense	Renal neoplasm	NSS Nephrectomy	3/LR 4/NED
12	2017	Moreillo-Vicente *et al*. [5]	61	M	Flank pain	R	40	Hilar	CT: heterogeneous mass with necrotic core MRI: T1 hypointense T2 hyperintense	Adrenal neoplasm	Nephrectomy	6/NED
13	2018	Our Case	61	M	Incidentally	L	38	Midportion	US: hypoechoic mass CT: peripheral enhancement MRI: peripheral enhancement	Renal neoplasm	Nephrectomy	108/NED

*CT* computed tomography, *F* female, *G* graft, *L* left, *LR* local recurrence, *M* male, *MRI* magnetic resonance imaging, *NA* not assigned, *NED* no evidence of disease, *NSS* nephron-sparing surgery, *R* right, *US* ultrasonography

## Case presentation

We describe a 61-year-old Arab man who retired from teaching 2 years ago. He did not smoke tobacco or consume alcohol. His past medical history included two surgical operations: a hydatid cyst of the liver operated on 6 years ago in a surgery department and a right ureteral lithiasis operated on in our urology department 4 years ago (at that time, he had only been explored by an intravenous pyelogram). He had been under alpha blocker for benign prostatic hyperplasia for 6 months. He was admitted for a suspicious renal cyst, incidentally found on renal and vesicoprostatic ultrasound. He had no complaints. His physical examination was unremarkable. His temperature was 37.2 °C, his blood pressure was 134/82 mmHg, and his pulse rate was regular at 74 beats per minute. On laboratory values, white blood cell count was 7.9 × 10^3^/mL, red blood cell count 4.1 × 10^6^/mL, hemoglobin 14.2 g/dL, platelets 396 × 10^3^/mL, creatinine 1.04 mg/dL, sodium 138 mEq/L, potassium 4.1 mEq/L, and C-reactive protein 1 mg/L. Urines examination showed no leukocyturia or bacteriuria.

Renal and vesicoprostatic ultrasound found a non-vascularized cystic formation with a thickened and irregular wall on his left kidney.

**Fig. 1:**
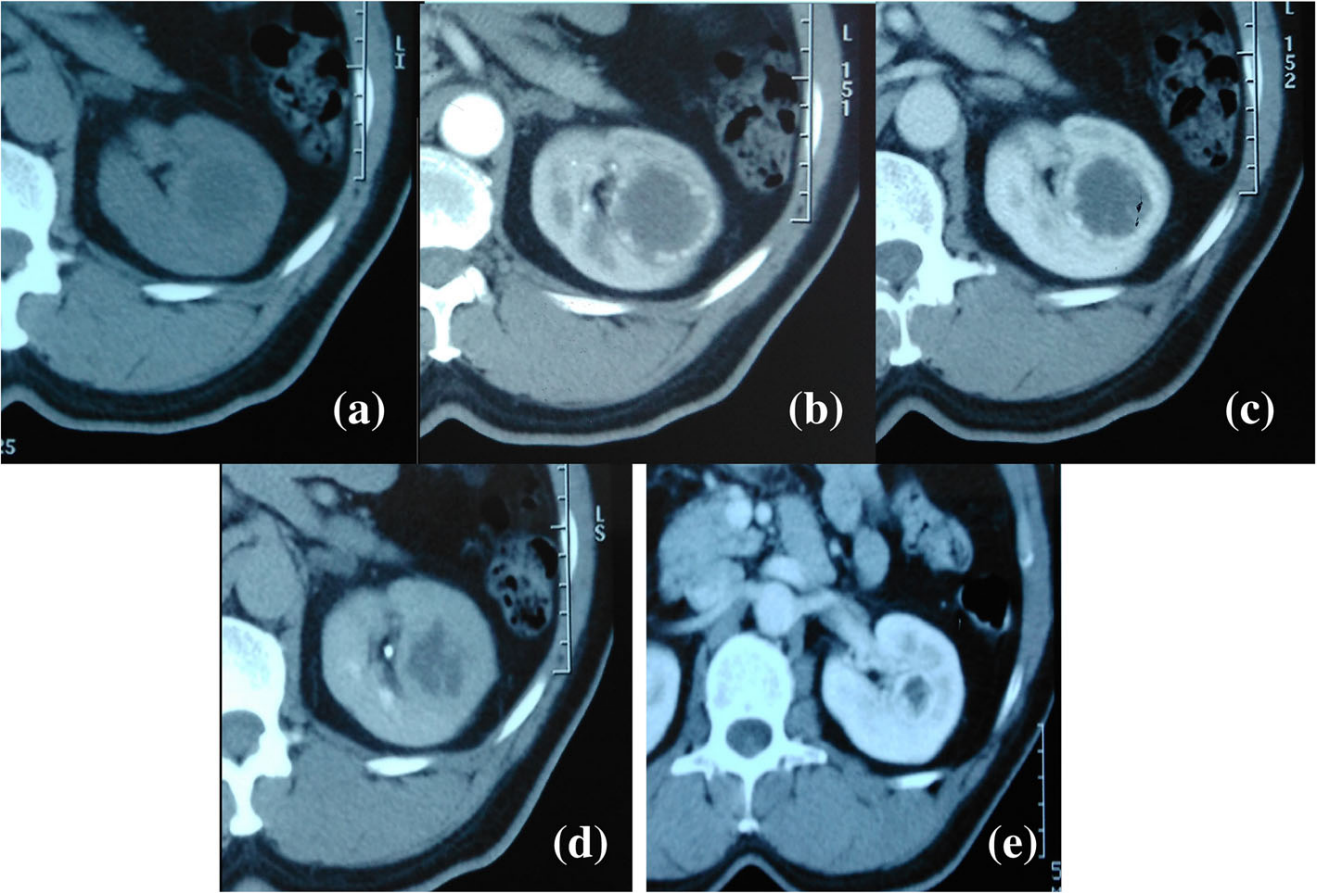
Computed tomography axial view of the tumor shows intense peripheral enhancement. **a** Non-enhanced computed tomography. **b** Arterial phase. **c** Portal phase. **d** Delayed phase. **e** The same lesion, 6 years ago

**Fig. 2:**
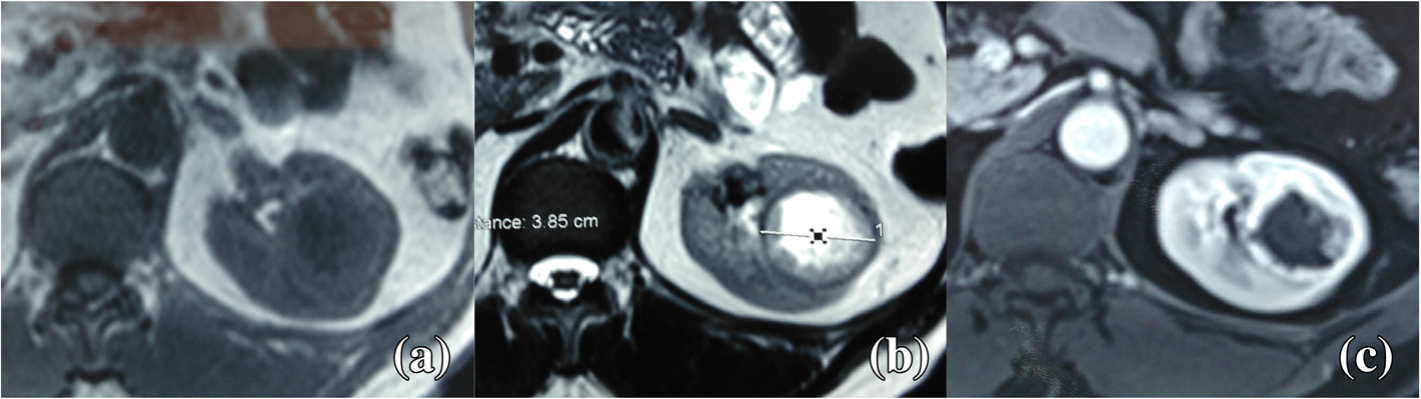
Magnetic resonance imaging axial view shows a hypointense mass on T1 (**a**) and hyperintense on T2 (**b**) with intense peripheral enhancement (**c**)

An abdominal computed tomography (CT) scan revealed a 38 mm left renal mid-pole lesion, isodense to the renal parenchyma. Dynamic CT showed an early intense and peripheral enhancement and nonenhanced central zone even in the delayed phase (Fig. 1). The renal artery and vein appeared normal. No metastases were demonstrated. We also recovered a CT realized 6 years ago in the surgery department, which illustrated the same lesion but 10 mm smaller (Fig. 1). For further characterization of the cyst, a magnetic resonance imaging (MRI) was performed. It revealed a lesion with thickened and irregular wall (from 3 to 10 mm) isointense on T1-weighted images and hypointense on T2- weighted images with intense enhancement. The central zone was hypointense on T1 and hyperintense on T2 with no enhancement (Fig. 2). Radiological findings concluded a Bosniak IV cyst. As this cyst type is considered clearly malignant, our patient was accordingly scheduled for surgery. A partial nephrectomy was considered technically difficult for this lesion, so he underwent an open left radical nephrectomy. His postoperative course was uneventful.

**Fig. 3:**
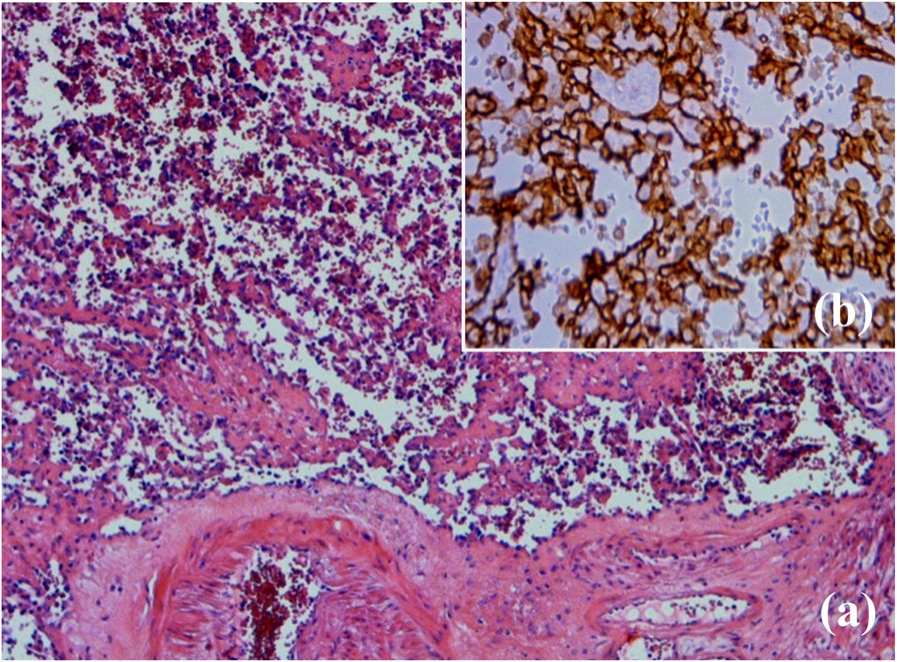
**a** The tumor composed of papillary and anastomosing channel-like structures (hematoxylin and eosin × 100). **b** Diffuse staining for CD31 (immunohistochemical × 200)

On gross examination, cut sections divulged a well-defined medio-renal hemorrhagic and brownish mass measuring 3 × 2.5 cm. Histological examination of the mass showed a mesenchymal proliferation arising from the wall of a large vessel and developing within its lumen. It was composed of hyalinized papillary and anastomosing channel-like structures that were lined by flat to plump endothelial cells with no atypia or mitotic activity (Fig. 3). An immunohistochemical study revealed diffuse staining of tumors cells for CD-31 and negativity for HMB-45 and cytokeratin (Fig. 3). The diagnosis of IPEH was retained. He was asymptomatic and no recurrence of the tumor has been detected during 9 years of regular clinical and radiological follow-up. Additional file 1 presents a timeline of the case.

## Discussion

Our case showed a Masson’s tumor of the kidney that presented as a suspicious cyst, with a slow growth over 6 years and 9 years of follow-up without recurrence. This is only the 12th renal case described in the literature (Table 1).

IPEH is a rare peculiar entity characterized by exuberant endothelial proliferation within the lumen of blood vessels [1]. Even though the precise etiology and physiopathology of IPEH remains undetermined and incompletely understood, many authors suggested that it could be linked to an alteration in the thrombosis process, due to an unusual thrombus organization [2]. Three types of IPEH have been defined: primary or pure type arising from dilated vessels; secondary or mixed type developing in pre-existing vascular lesions such as hemangioma; and third or extravascular type originating from hematomas [1]. In the kidney, IPEH can occur within vessels at many levels: the renal vein, the renal sinus, or the renal parenchyma per se [1]. IPEH has a frequent association with thrombus [2], but not in our case or in three others [1, 3, 4].

IPEH was mainly reported in the extremities skin and soft tissues. Solid organs have been rarely involved [5]. The first renal case was described by Garber *et al.* [6] in 1990 as a new renal lesion. IPEH generally occur at any age and most often in female patients [5]; however, a renal location seems to be involved more frequently in adult males than females (Table 1). Two cases were reported in patients with chronic kidney failure [7, 8]. The clinical manifestation of this tumor is not specific and varies widely. It can produce, as any other renal mass, flank pain, hematuria, or massive retroperitoneal hemorrhage or it can be asymptomatic and fortuitously diagnosed, as in our case. Lesion size ranges from 18 to 55 mm and is mostly localized in the left kidney.

In soft tissues, ultrasound shows typically a hypoechoic lesion and dynamic CT shows high peripheral enhancement of the lesion [9]. In the kidney, radiological features are non-specific to differentiate IPEH from other suspicious renal masses. However, in all cases, Masson’s tumor is located in the renal hilum or in the midportion of the kidney (Table 1), which should be considered in the diagnoses.

Preoperative diagnosis of renal IPEH was hard to carry out and this led to nephrectomy in all cases. It was managed by a partial nephrectomy as mentioned in three cases [1, 3, 10]. In other cases, radical nephrectomy was realized since tumors were located in the renal hilum.

Through Table 1, we can see that some features could evoke the diagnosis of renal IPEH. In that case, a lesion biopsy should be realized and the kidney could be spared. In our case, first, even though the slow growth of the tumor suggested a benign lesion, our decision to proceed in surgery was influenced by the radiological findings that indicated a Bosniak IV cyst, which is malignant in more than 90% of cases [11]. Second, we did not consider a biopsy because it was not recommended for cystic masses in the guidelines of that time [11].

Finally, neither metastases nor malignant degeneration has been reported with renal IPEH. There is only one case of recurrence, which occurred after a nephron-sparing surgery [10].

## Conclusions

Masson’s tumor is a benign vascular degeneration. A renal localization for Masson’s tumor could barely be found in the literature. Preoperative diagnosis can be a real challenge. Nephrectomy was realized in all cases because of this entity’s non-specific radiological characteristics among suspicious renal masses.

Our case showed that Masson’s tumor can present as a suspicious renal cyst, an aspect that was not previously described in the few cases reporting this process in the kidney, and our literature review confirms that some features might evoke the diagnosis. Thus, this entity should be considered more often in the thick of the diagnostic possibilities in order to avoid unnecessary nephrectomies.

## Additional file

Additional file 1: Timeline of the case. (DOCX 14 kb)

## Abbreviations

CT: Computed tomography
IPEH: Intravascular papillary endothelial hyperplasia
MRI: Magnetic resonance imaging
