# Supplementary material for: Masson’s tumor of the kidney: a case report
Source: J Med Case Rep. 2018 Dec 22;12:376. doi: 10.1186/s13256-018-1898-2 (PMC6303989; doi:10.1186/s13256-018-1898-2)
Supplement: Supplementary file 1 — Timeline of the case. (DOCX 14 kb) [file 13256_2018_1898_MOESM1_ESM.docx]

**A male patient, born in 1948**

**2003**

Operated for an hydatid cyst of the liver

**2005**

Operated for a lithiasis of the right ureter

A renal complex cyst incidentally found on an ultrasound

Physical exam: No abnormalities

CT/MRI: Bosniak IV cyst

Open Right Nephrectomy

Pathological result:
Masson tumor of the kidney

**2009**

**2010
2017**

Patient seen once a year, with creatinine and abdominal ultrasound result

**-**

No complaint
Creatinine: 1.19 mg/dl
Abdominal ultrasound: No abnormalities

**2018**
